# Selection at the *Esterase-2* Locus of *Drosophila buzzatii*? Perturbation-Reperturbation Experiments

**DOI:** 10.1371/journal.pone.0108147

**Published:** 2014-09-24

**Authors:** J. Stuart F. Barker, Peter C. Thomson

**Affiliations:** 1 School of Environmental and Rural Sciences, University of New England, Armidale, NSW, Australia; 2 ReproGen, Animal Bioscience Group, Faculty of Veterinary Science, The University of Sydney, Camden, NSW, Australia; University of Arkansas, United States of America

## Abstract

Apparent selection affecting starch gel electrophoretic alleles at the *Esterase-2* locus of *Drosophila buzzatii* has been detected in laboratory and natural populations. Perturbation-reperturbation of allele frequencies in replicated laboratory populations attempts to test direct selective effects at the locus versus effects of linked loci. Sequential gel electrophoresis has identified more alleles within starch classes, and three of these alleles (within the *a*, *b* and *c* starch alleles) were used in cage population experiments. Allele *a/1.00/1.00/1.00* was set up in 10 replicate populations with allele *c/1.00/1.00/1.00*, and in an independent 10 replicate populations with allele *b/0.99/1.01/1.00*. For each set, three reperturbations were done. Replicate populations generally showed similar patterns of allele frequency change and clear directionality: effects of selection, not drift. However, four populations deviated from their replicates, indicating dissipation of linkage disequilibrium. Estimates of pre-adult viability in the F_2_ of pair-wise crosses among 12 sequential gel electrophoretic alleles showed very variable modes of inheritance and relative viability fitnesses. Together with the diversity of patterns of allele frequency change in the cage populations, these results suggest a gene complex, with selection acting on an interacting set of loci which may include *Esterase*-2.

## Introduction

The determination of selective differences among genotypes at a single locus has long remained as an outstanding problem in population genetics [1]–[3], with the problem more recently expressed in terms of the genetics of adaptation [4], [5]. The alternative hypotheses of neutrality or selection [1], [6], [7] stimulated major debate, and although the former is the appropriate null hypothesis, the problem is not just to distinguish these alternatives. Firstly, selective differences among genotypes may be present and important on an evolutionary time scale, but be too small to be detected experimentally. Secondly, selection may be detected as affecting allele and genotype frequencies at the locus, but that is not sufficient, as it does not demonstrate that the effects are the direct result of fitness differences among genotypes at that locus. Rather the effects may be due to linked loci. Perturbation-reperturbation experiments in laboratory populations [8] offer the possibility of distinguishing direct effects at the locus from effects of linked loci.

The rationale for a perturbation experiment, either in natural or laboratory populations, is to perturb allele frequencies away from putative equilibrium frequencies, and monitor subsequent changes. If the putative equilibrium frequencies are due to some form of balancing selection, allele frequencies will change back to that equilibrium. However, if the variation at the locus is neutral, allele frequencies will change due to drift, but the direction of change will be indeterminate. Árnason [8] gave the criteria for an appropriate perturbation experiment as: (i) replication of populations – as changes due to drift may seem directional in a single population over a short time scale, (ii) initiation of populations at both high and low starting allele frequencies – as balancing selection predicts an internal equilibrium, this equilibrium should be approached from both above and below, and (iii) combining a number of independently derived lines carrying the alleles – so that initial linkage disequilibria will be minimised by randomising variation at the locus relative to background variation. Given these criteria, drift or selection affecting allele frequencies may be distinguished. However, if selection is implicated, a follow-up reperturbation experiment is necessary to attempt to distinguish direct selection at the locus from effects of linked loci (linkage disequilibria).

In the perturbation- reperturbation experiments reported here, we have taken a different approach to Árnason’s criterion (iii), and used isogenic lines, with potentially strong linkage disequilibrium in the region around the studied locus. If all replicate populations, both initial and reperturbed, approach the same internal equilibrium or fixation of one allele, then selection is implicated. However, it may be selection at the studied locus itself, or selection acting on one or more other loci that are in strong linkage disequilibrium with the studied locus. But replicates deviating from the common pattern of allele frequency change indicate partial or complete dissipation of the disequilibrium, and prove that linkage effects are involved. Repeatable directional changes in allele frequencies in replicates following reperturbation, and which tend to the same internal equilibrium (or fixation) as the initial cages indicate no dissipation of linkage, while changes to a different internal equilibrium may be due to partial or complete dissipation, or to the generation of new linkage disequilibria following the reduction in population size at the reperturbation. One or more of the above outcomes implicate effects of linkage and selection directed at other loci. On the other hand, if no breakdown of linkage disequilibrium is observed, selection is directly at the marker locus or for one or more tightly linked genes, or a block of tightly linked and possibly interacting genes.

The *Esterase-2* locus of *Drosophila buzzatii* is highly polymorphic, with five common (designated *a*, *b*, *c^+^*, *c* and *d*) and three rare electromorphs (designated a*, *e* and *null*) detected using starch gel electrophoresis (starch GE) [9], [10] and 25 alleles using sequential gel electrophoresis (sequential GE) [11]. There is extensive evidence, from both natural populations and laboratory experiments, that selection is affecting the frequencies of the starch GE alleles, and consequently that variation at this locus may be adaptively significant:

(i). Coincident patterns in Australia and Argentina of clinal (latitudinal) variation in allele frequencies, which could not be accounted for by linked inversions [12], [13],
(ii). Perturbation experiments in natural populations [14], [15],
(iii). Significant genotype-environment associations in Australia [16]–[18] and in Argentina [13],
(iv). Differential attraction to and viability on diverse cactus hosts [19],
(v). Genotypes differentially attracted to cactus specific yeast species [20], [21],
(vi). Responses of adults to thermal shocks [22],
(vii). Biochemical and physiological analyses [23] and molecular analyses [24] indicate that selection may be acting directly at the locus, and
(viii). For four sequential GE alleles, significant viability and developmental time differences following pupal heat stress [25].

We report here results of two perturbation-reperturbation experiments, using the most common sequential GE allele in the ‘*a*’ starch class with each of two other sequential GE alleles (one in the ‘*b*’ and one in the ‘*c*’ starch class), and of an experiment to estimate relative pre-adult viabilities from pair-wise tests of 12 of the sequential GE alleles. The results indicate that selection is acting on an interacting set of loci that may include *Esterase*-2.

## Material and Methods

### Isochromosomal lines

Isofemale lines of *D. buzzatii* were established from collections at two *Opuntia stricta* Haworth sites - Trinkey, New South Wales (31° 22'S, 149° 27'E) in December, 1984, and Hemmant, Queensland (27° 26'S, 153° 08'E) in November, 1985. From one male in each isofemale line, and using the *Antennapedia*/*Delta 5* (*Antp*/Δ*5*) second chromosome balancer stock, one line was derived which was isochromosomal for a wild chromosome 2, with the Y chromosome from the original wild male, chromosomes 3 and 4 from the original wild male and from the *Antp*/Δ*5* stock, and X chromosomes from the *Antp*/Δ*5* stock (see [11] for details).

From Trinkey, 71 such lines were obtained, and 76 from Hemmant. As the *Est-2* locus maps close to the proximal break points of the two more common inversions [26], [27], all lines were karyotyped for chromosome 2 sequences [11]. Of the Trinkey lines, 21 were *ST*, 46 *j* and four *jz^3^*, and the corresponding numbers for Hemmant were 20, 55 and one. All lines were characterised for their *Est-2* genotype using sequential gel electrophoresis [11]. During the period of karyotyping and electrophoretic characterisation, all lines were maintained in four vials, five pairs per vial.

### Cage fitness experiments

Three lines, all in the *j* inversion background, were chosen for the cage fitness experiments. The allele in each line was that at the highest frequency in each of the *a*, *b* and *c* starch classes in the natural populations. These alleles (names as in [11]), line names and population frequencies (in the 147 pooled Trinkey (IT) and Hemmant (IH) lines) were:

*a/1.00/1.00/1.00*   IT6    0.279

*b/0.99/1.01/1.00*   IT15   0.075

*c/1.00/1.00/1.00*   IH13   0.082

IT6 was taken arbitrarily as the standard line, and each of IT15 and IH13 were backcrossed to IT6 males for 25 generations. Full details of the backcrossing procedures are given in Document S1(a). The resulting isogenic lines, which thus have very similar genetic backgrounds, except for a region averaging four map units (100/25 [28]) on either side of the *Est-2* locus, were used to initiate population cages for the perturbation experiments. The lines were set up in two combinations – in one cage for IT6/IT15 (starch GE alleles *a* and *b*) and one for IT6/IH13 (starch GE alleles *a* and *c*). Details of initial cage setup are given in Document S1(b).

Eggs were collected from these cages to start independent replicate populations that derived from the same foundation population. Five food cups introduced into each cage on Mondays, Wednesdays and Fridays were removed when the next cups were introduced, and one was placed into each of five replicate cages. This procedure was repeated for three weeks to give nine food cups in each of the replicate cages. Cages then were maintained with cup replacement on a regular Monday, Wednesday, Friday schedule. Table 1 gives the identification numbers of all cages, and the treatment for each. A first egg sample to estimate foundation allele frequencies was taken two days after adults were put in the egg laying cages. For IT6/IH13, egg samples were taken at 30 day intervals to day 90, then every 70 days to day 1350, with two further samples on days 1630 and 1910. IT6/IT15 cages were sampled at 35 day intervals to day 105, then every 70 days to day 1085, when some cages were terminated. The remaining cages were sampled irregularly to day 2065.

**Table 1:** Cage identification numbers, days from set-up of initial cages and description.

Cage ident. no.	Days from set-up	Description
**IT6/IH13 a/c**		
1–5	0	Initiated at low frequency of IT6(a) allele
6–10	0	Initiated at high frequency of IT6(a) allele
1R1 – 5R1	656	First reperturbation from Cages 1–5 respectively, to decrease frequency of IT6(a) allele
2R2, 5R2	1819	Second reperturbation –from Cages 2R1 and 5R1 to decrease frequency of IT6(a) allele
2R3	2301	Third reperturbation – **again** from Cage 2R1 to decrease frequency of IT6(a) allele
**IT6/IT15 a/b**		
11–15	0	Initiated at low frequency of IT6(a) allele
16–20	0	Initiated at high frequency of IT6(a) allele
16R1 – 20R1	552	First reperturbation from Cages 16–20 respectively, to increase frequency of IT6(a) allele
16R2 – 20R2	1247	Second reperturbation from Cages 16R1 – 20R1 respectively, to increase frequency of IT6(a) allele
16R3 – 19R3	2189	Third reperturbation - **again** from Cages 16R1 – 19R1 respectively, to increase frequency of IT6(a) allele

### Reperturbation

For each cage, egg samples were taken by inserting an extra food cup for 24 h, then transferring sections of the medium surface to six bottles, and repeating one day later. Virgin emergences were collected from the 12 bottles for each cage, stored for 3 days, and about 200 pair matings set up. After two days, parents were removed and frozen for electrophoresis. For IT6/IT15, the IT6 (*a*) allele generally decreased in frequency, so reperturbation involved increasing the frequency of this allele. Thus vials where parents were *aa* × *aa*, *aa* × *ab* or *ab* × *ab* (*a*  =  IT6 allele) were identified and eight to 11 vials placed in each new cage. For IT6/IH13, the IT6 (*a*) allele generally increased in frequency, so reperturbation involved decreasing its frequency (increasing the *c* allele frequency). Thus vials where parents were *cc* × *cc*, *ac* × *cc* or *ac* × *ac* (*c* – IH13 allele) were identified and 10 to 18 vials placed in each new cage. The allele frequency at the initiation of each replicate reperturbation cage was estimated from the genotypes of the parents in the vials used for that cage. One food cup was put into each cage the day before progeny started emerging from the vials, and the three food cups per week schedule then followed. Table 1 gives the schedule of reperturbations and the identification numbers for each cage. Subsequent reperturbations used the same procedure, with the number of vials added to each cage ranging from nine to 17.

### All cages – allele frequency estimation and cage maintenance

Egg samples to estimate allele frequencies were taken by placing an extra food cup in a cage for 24 hours, then putting sections of the medium surface into each of 10 vials to allow development under uncrowded conditions. All emerging adults were collected (usually daily), pooled across vials and stored frozen for later electrophoresis. Thirty-six flies of each sex were electrophoresed from each sample, with numbers from each day of emergence proportional to numbers collected that day.

During all backcrossing and for the cage samples, procedures for starch GE of *Est-2* were as in [9], with modifications [10], [17]. All flies throughout line isolation and in the cages were cultured on autoclaved sucrose-yeast-cactus medium [29], seeded with live *Saccharomyces cereviseae*, and kept at 25±0.5°C, 65–70% relative humidity and a light cycle of L12:D12 (light 0700 – 1900). The population cages (29×38×20 cm) were constructed of galvanised iron, and the cage cups were disposable 200 ml plastic cups with 50 ml medium (approx. 28 cm^2^ surface area).

### Statistical analyses

Time course analysis was used to compare the patterns of allele frequency change for three data sets: (1) IT6/IH13, cages 1–5 vs cages 6–10, (2) IT6/IH13, cages 1–5 vs cage 2R1, and (3) IT6/IT15, cages 11–15 (excluding cage 13) vs cages 16–20 (excluding cage 19). To allow for the nonlinear changes in the allele frequency over time, a spline smoothing approach was adopted. The following linear mixed model was fitted to each set of data:
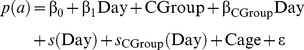
where *p*(*a*)  =  allele *a* frequency; Day  =  number of days (fixed covariate effect); CGroup  =  effect of group of cages for comparison (fixed factor effect); Cage  =  effect of Cage (random effect); and ε  =  random error. Further, β_0_  =  intercept; β_1_  =  overall linear regression slope for Day; β_CGroup_  =  adjustment to linear regression slope for Day for the cage group; (i.e. Day × CGroup interaction in linear trend); *s*(Day)  =  overall smoothing spline function of Day; and *s*_CGroup_(Day)  =  smoothing spline function of Day for the cage group (i.e. Day × CGroup interaction in spline). To account for serial correlation in the data, an exponential correlation structure was included in the specification of the random errors for repeated measurements on the same cage. Fitting of the models was conducted in ASReml-R. Note that for fitting splines in this package, it is necessary to fit linear trends as fixed effects, and the splines (nonlinearity) fitted as random effects.

Tests for CGroup × Day interactions (to assess different shaped time courses for each group of cages) were conducted by combining test statistics for both linear and nonlinear (spline) test statistics. The test for the linear trends was assessed by Wald chi-squared tests, and the tests for the nonlinear splines by REML likelihood ratio tests (full vs reduced models).

Model predictions were obtained over a range of values of Days consistent with observed data values. Model-based cage-group means were obtained at specific time points, 1,910 days for Data Sets 1 and 2; 1155 and 1645 days for Data Set 3, and these means were compared using approximate *z*-tests.

### Viability fitness experiment

At the same time that the isochromosomal lines IT15 and IH13 were backcrossed to IT6, nine other isochromosomal lines were backcrossed to IT6, also for 25 generations. All were in the *j* inversion background. These lines, which cover the range of sequential GE alleles (Table 2), were used in a series of pair-wise crosses which were taken through to the F_2_ to test segregation ratios. For each cross, two bottles (25 pairs per bottle) were set up for each reciprocal. The progeny from each reciprocal cross were used to set up two bottles (25 pairs per bottle). Progeny of these F_1_ matings were collected every one or two days, sexed, counted and frozen in Eppendorf tubes. From each F_1_ mating, a random sample of 72 males and 72 females matching the emergence distribution was set up for starch GE, or 48 males and 48 females when cellulose acetate electrophoresis was necessary to distinguish the alleles. Observed numbers of each genotype were compared to the expected 1∶2∶1 ratio, and significance tested using chi-square, separately for each sex in each reciprocal cross and overall. For the significant pair-wise crosses, observed progeny numbers were expressed as relative viability fitness.

**Table 2:** Allele and line designations of 12 lines tested in pair-wise combinations for pre-adult viability fitness, and frequency of each allele in the 147 lines derived from two populations (Barker 1994).

Electrophoretic allele	Line designation	Allele frequency
*a 1.04/0.98/1.09*	IT42	0.027
*a 1.02/1.00/1.04*	IH4	0.061
*a 1.00/1.00/1.00*	IT6	0.272
*b 1.00/1.02/1.00*	IH25	0.034
*b 1.00/1.01/1.00*	IT64	0.027
*b 0.99/1.01/1.00*	IT15	0.075
*b 0.99/1.00/0.96*	IT16	0.020
*c 1.00/1.00/1.00*	IH13	0.068
*c 0.98/0.99/0.98*	IT8	0.034
*c^+^ 1.00/1.00/1.00*	IH31	0.075
*d 1.00/0.98/1.00*	IT49	0.041
*d 0.97/1.00/0.92*	IT46	0.027

In fulfillment of data archiving guidelines, all data are available from the Dryad Digital Repository: http://doi.org/10.5061/dryad.hq347.

## Results

### Cage fitness experiments - IT6/IH13 (starch GE alleles a/c)

**Figure 1:**
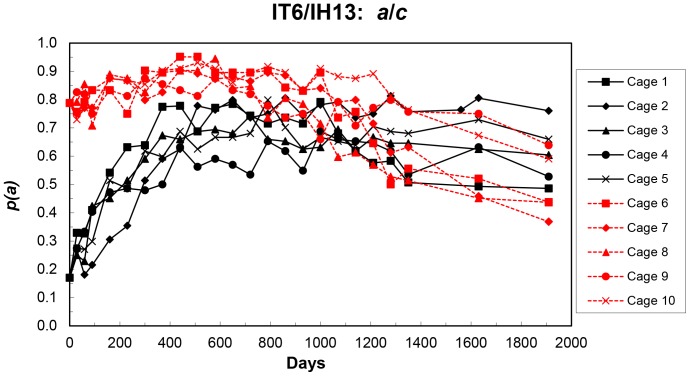
Frequencies of the IT6 (*a*) allele in each replicate cage at each sampling of the initial IT6/IH13 populations.

**Figure 2:**
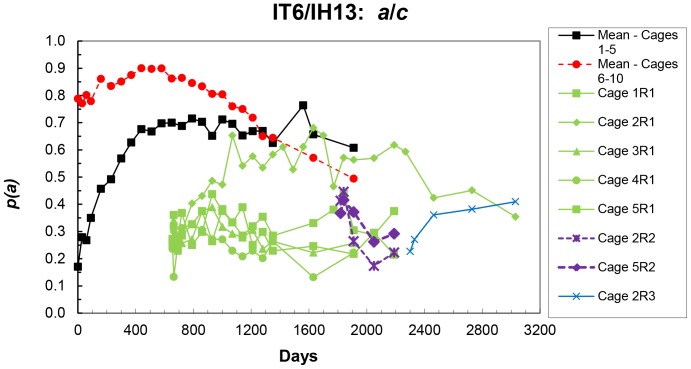
Mean frequencies of the IT6 (*a*) allele in the initial populations, and frequencies in each replicate cage of the first reperturbation (R1), the second (R2) and the third (R3) at each sampling – IT6/IH13 populations.

All 10 cages showed an initial increase in the frequency of the *a* allele (hereafter *p*(*a*)), with high repeatability of allele frequency changes within each set of the low and high initial frequency cages (Figure 1). The means over replicate cages of each set (Figure 2) show the five 0.25 initial frequency cages (cages 1–5) possibly at equilibrium from day 510 to day 1910 at an average *p*(*a*) of 0.674±0.032. For the five 0.75 initial frequency cages (cages 6–10), *p*(*a*) increased to about day 580, and then decreased to day 1910, when all ten cages were terminated. Time course analysis (Figure S1 in Document S1) showed *p*(*a*) in both cages 1–5 and 6–10 to be decreasing from peak values. The rate of decrease was greater for cages 6–10, and the predicted *p(a)* values at day 1910 (cage termination) were significantly lower (*P* = 0.026) than for cages 1–5 (Figure S1 in Document S1).

Each of cages 1–5 were reperturbed, reducing *p(a)* to an average of 0.257±0.015 at day 656 in cages 1R1 to 5R1 (Figure 2). Four of these five cages (except for cage 2R1) then showed essentially no change in *p*(*a*) (Figure 2), with an average frequency to day 2190 of 0.287±0.032. For cage 2R1, however, *p*(*a*) increased to day 1070 at essentially the same rate as the initial low frequency cages, and then remained stable to day 2268 (average *p*(*a*) over this period of 0.585±0.054; with a predicted value at day 1910 not significantly different (*P* = 0.85) from that for cages 1–5 (Figure S2 in Document S1). The frequency then decreased to an average of 0.410±0.050 over the period days 2470–3030.

The two cages (2R2 and 5R2) of the second reperturbation (derived from cages 2R1 and 5R1) had a higher initial frequency (0.390±0.032 at day 1819) than those of the first reperturbation, and *p*(*a*) decreased rapidly in both to values similar to the average for the R1 cages (except 2R1), i. e. to mean frequencies of 0.218 and 0.257 at days 2050 and 2190 respectively (Figure 2). Cage 2R1 was perturbed again for a third reperturbation (to produce cage 2R3 at day 2301), and *p*(*a*) in cage 2R3 increased to a value similar to that of cage 2R1 at day 3030 (Figure 2), when the cages were terminated.

Over all the IT6/IH13 cages, the *a* allele frequencies show three putative equilibria: (i). about 0.6 for the 10 initial cages and transiently for cage 2R1, (ii). about 0.3 for the cages of the first reperturbation (except 2R1) and for the two cages of the second reperturbation (2R2 and 5R2), and (iii). about 0.4 for cage 2R1 after day 2400, and for cage 2R3.

### Cage fitness experiments - IT6/IT15 (starch GE alleles a/b)

**Figure 3:**
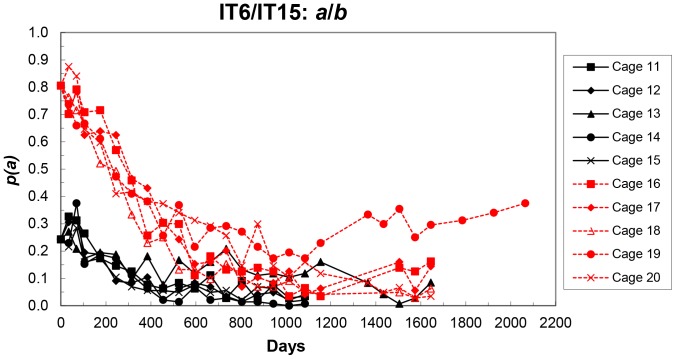
Frequencies of the IT6 (*a*) allele in each replicate cage at each sampling of the initial IT6/IT15 populations.

All 10 cages showed an initial decrease in *p*(*a*) and except for cages 13 and 19, high repeatability of allele frequency changes (Figure 3). In the 0.25 initial frequency cages (cages 11–15, except cage 13), *p*(*a*) decreased to an apparent equilibrium over days 735-1085 (mean over cages  = 0.033±0.011). However, this apparent equilibrium may simply reflect the expected low rate of change at low allele frequency. These cages were terminated after the day 1085 sampling. The initial trend of change in *p*(*a*) in cage 13 was the same as in the other four cages to day 315, but for days 315-1155, the frequency remained at an apparent equilibrium (mean  = 0.135±0.038). *p*(*a*) then fluctuated until cage termination at day 1645, but over this period average *p*(*a*) was very similar to that for the 0.75 initial frequency cages (16, 17, 18 and 20).

The 0.75 initial frequency cages showed a similar pattern – one aberrant cage (cage 19) and four with a similar initial decrease in *p*(*a*) to an apparent equilibrium over days 805-1645 (mean over cages  = 0.096±0.028). Time course analysis showed *p*(*a*) in cages 16–20 (excluding cage 19) was not significantly different from that in cages 11–15 (excluding cage 13) at day 1155 (*P* = 0.23) nor at day 1645 (*P* = 0.98), as well as any intervening days (Figure S3 in Document S1). Further, cage 13 actual values were within the range of values for cages 16–20 (excluding cage 13) at day 1645. The frequencies of *p*(*a*) in cage 19 were similar to those in the other four cages to day 595, but subsequently remained above their average frequency. Up to day 1085, the frequency in cage 19 was not markedly different from the other cages, but it then increased to a possible equilibrium over days 1365–2065 (mean  = 0.320±0.039).

**Figure 4:**
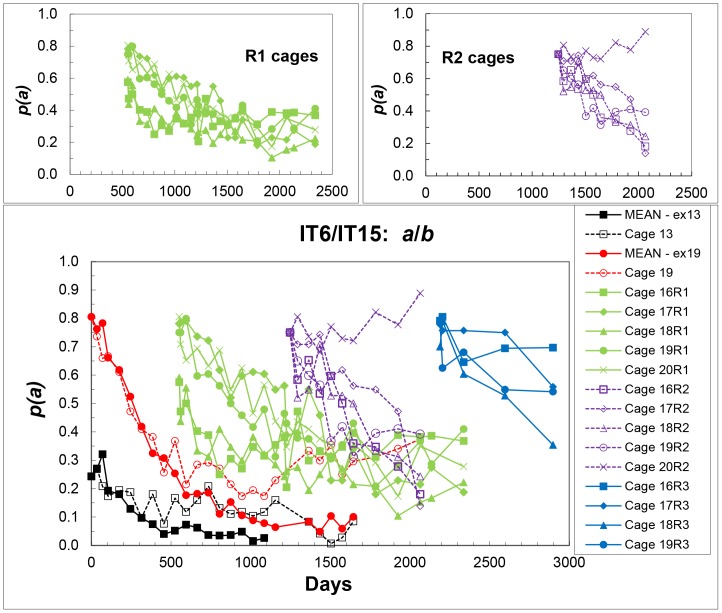
Mean frequencies (excluding cages 13 and 19) of the IT6 (*a*) allele in the initial populations, frequencies in cage 13 and in cage 19, and frequencies in each replicate cage of the first reperturbation (R1), the second (R2) and the third (R3) at each sampling – IT6/IT15 populations. For clarity, R1 and R2 results are also given separately in the small plots.

**Figure 5:**
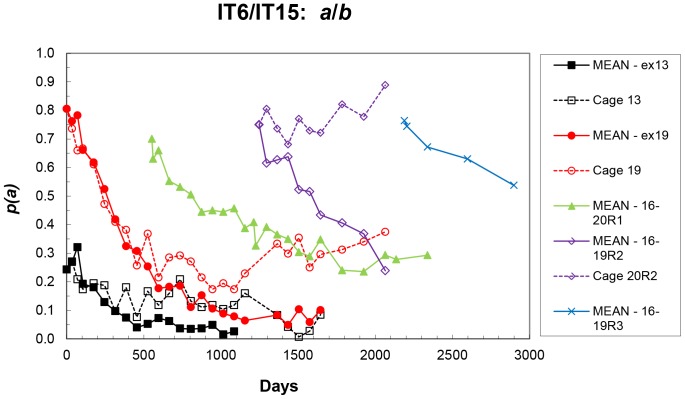
Mean frequencies of the IT6 (*a*) allele in the initial populations (excluding cages 13 and 19), mean frequencies of the first reperturbation (R1) populations, of the second (R2-excluding 20R2) and the third (R3), and frequencies in cages 13, 19 and 20R2 at each sampling – IT6/IT15 populations.

Summary results for these cages (mean – cages 11–15, excluding cage 13; cage 13; mean – cages 16–20, excluding cage 19; cage 19, and all reperturbation cages) are shown in Figure 4, and as replicate means for all sets in Figure 5.

Although the initial frequencies of the first reperturbation cages (16R1-20R1) were variable (Figure 4), the patterns of allele frequency change in the five cages were very repeatable, with no significant trend in the CV of cage means. However, the rate of allele frequency change is slower than in the set of initial 0.75 allele frequency cages, and *p*(*a*) appears to be reaching an internal equilibrium of about 0.3. Cage 19 (aberrant cage of the 0.75 initial frequency set) deviates from the other cages of this set, but then attains possibly the same equilibrium as the R1 cages. Cages 16R2–19R2 of the second reperturbation were derived from the R1 cages, and also may have been approaching this same equilibrium, but unfortunately they were discarded too early to be certain. Cage 20R2 clearly was aberrant in this set, with a trend of increasing frequency. Cages 16R3 – 19R3 (also derived from 16R1-19R1) also show a decreasing trend in *p*(*a*), but were not maintained for long enough to detect definite trends. Except for the three cages 13, 19 and 20R2, each set of the initial and reperturbation cages shows high repeatability in the patterns of change in *p*(*a*) (Figures 4 and 5).

### Viability fitness experiment

Of the 66 possible crosses, 48 were completed, with no significant differences in the results for any of the reciprocal crosses. Table 3 lists the crosses that showed a significant deviation from the expected 1∶2∶1 ratio (22), those that were not significant (26) and those that were not tested (18). For those that were significant, Table 4 gives the estimated relative pre-adult viabilities.

**Table 3:** All pair-wise crosses, showing those that were not tested (X), those not significant (ns), and results of significance tests for the remaining 23.

Line	IT42(*a*)	IH4(*a*)	IT6(*a*)	IH25(*b*)	IT64(*b*)	IT15(*b*)	IT16(*b*)	IH13(*c*)	IT8(*c*)	IH31(*c^+^*)	IT49(*d*)
IH4(*a*)	X										
IT6(*a*)	ns	**									
IH25(*b*)	***	*	ns								
IT64(*b*)	ns	ns	*	X							
IT15(*b*)	*	* f	***	X	* m						
IT16(*b*)	***	**	ns	X	X	X					
IH13(*c*)	ns	ns	ns	ns	ns	ns	X				
IT8(*c*)	***	ns	ns	ns	***	ns	***	X			
IH31(*c^+^*)	X	X	ns	X	X	*	X	X	X		
IT49(*d*)	***	ns	ns	* m	*	ns	***	ns	** m	X	
IT46(*d*)	ns	***	ns	ns	ns	ns	***	* f	* m	X	X

Among the latter, f indicates significance for females only and m significance for males only.

*P<0.05,

**P<0.01,

***P<0.001.

**Table 4:** Relative pre-adult viabilities of the three genotypes expressed in the F_2_ for 23 line crosses.

Lines		Relative viabilities			Mode^a^
1	2	11	12	22	
IT42 (*a*)	IH25 (*b*)	1.00	0.17	0.22	rec +
IT42 (*a*)	IT15 (*b*)	1.00	0.68	0.71	rec +
IT42 (*a*)	IT16 (*b*)	1.00	0.30	0.24	rec +
IT42 (*a*)	IT8 (*c*)	1.00	5.65	2.10	OD
IT42 (*a*)	IT49 (*d*)	1.00	0.43	3.78	?
IH4 (*a,104*)^b^	IT6 (*a, 100*)	1.00	0.87	1.51	dom −
IH4 (*a*)	IH25 (*b*)	1.00	0.82	0.71	rec +
IH4 (*a*)	IT15 (*b*) f^c^	1.00	1.67	1.19	OD
IH4 (*a*)	IT16 (*b*)	1.00	1.08	1.63	dom −
IH4 (*a*)	IT46 (*d*)	1.00	1.54	0.88	OD
IT6 (*a*)	IT64 (*b*)	1.00	1.03	0.68	dom +
IT6 (*a*)	IT15 (*b*)	1.00	1.13	1.59	dom −
IH25 (*b*)	IT49 (*d*) m	1.00	1.81	1.17	OD
IT64 (*b, 100*)	IT15 (*b, 99*) m	1.00	0.54	0.74	rec +
IT64 (*b*)	IT8 (*c*)	1.00	0.55	0.38	rec +
IT64 (*b*)	IT49 (*d*)	1.00	1.04	0.70	dom +
IT15 (*b*)	IH 31 (*c^+^*)	1.00	1.34	1.02	OD
IT16 (*b*)	IT8 (*c*)	1.00	0.99	0.44	dom +
IT16 (*b*)	IT49 (*d*)	1.00	1.11	0.58	dom +
IT16 (*b*)	IT46 (*d*)	1.00	0.95	0.48	dom +
IH13 (*c*)	IT46 (*d*) f	1.00	0.78	0.64	rec +
IT8 (*c*)	IT49 (*d*) m	1.00	1.95	2.30	rec −
IT8 (*c*)	IT46 (*d*) m	1.00	0.67	0.59	rec +

a Mode of inheritance expressed in terms of the first listed allele, where rec  =  recessive, dom  =  dominant (both of which may be partial), and +  =  allele 1 favoured, -  =  allele 1 selected against, OD  =  overdominance

b First part of sequential GE name is given when both alleles are from the same starch GE class

c f  =  significant deviation from 1∶2∶1 in females only, m  =  in males only.

## Discussion

In general, replicate populations – both initial and reperturbed – show very similar patterns of allele frequency change and clear directionality. There can be no doubt that selection is affecting *Est-2* allele frequencies. But equally clearly, the divergent populations – Cage 2R1 among IT6/IH13 and Cages 13, 19 and 20R2 among IT6/IT15 show that linked loci are involved and that recombination may dissipate linkage disequilibrium (at least partially), thus changing the selection regime. Further, a range of different apparent equilibria were observed:

(i). in the IT6/IH13 populations - one clearly transient (Cage 2R1, days 1070-2268) and two others - one for the initial cages 1–5 (about the same as the transient one for 2R1), and one for the R1 cages (except 2R1) and the R2 reperturbation cages (Figure 2),
(ii). in the IT6/IT15 populations – one for the initial cages and one for cage 19 and the R1 and R2 reperturbation cages (except 20R2).

These various equilibria, even if transient, show that a number of loci must be in linkage disequilibrium with *Est-2* in the initial populations, and involved in driving the selection. The apparent equilibrium at low *p*(*a*) observed for the initial cages 11–20 (except cage 19) may well be transient. In the pair-wise test for IT6 vs IT15, the IT6 (*a*) allele is dominant or semi-dominant for pre-adult viability and selected against (Table 4). If semi-dominant for overall fitness, the approach to *p*(*a*)  = 0 will be slow. In the IT6/IT15 populations, the different patterns of change in allele frequency between the initial and the three sets of reperturbation cages, whether due to breakdown of disequilibrium or generation of new disequilibria at reperturbation, also implicate multiple loci.

However, the high repeatability of some of the reperturbation populations may not seem to fit readily with this conclusion. For example, in the first reperturbation of the IT6/IH13 populations (Cages 1R1 – 5R1), *p*(*a*) in Cage 2R1 increased to an apparent equilibrium the same as that for Cages 1–5, i. e. no dissipation of linkage disequilibrium (Figure 2). The other four first perturbation populations show a different but highly repeatable pattern of stable allele frequency, presumably due to some dissipation of the disequilibrium. As there is no directionality in the changes in *p*(*a*) in these four populations, drift might be invoked, but *p*(*a*) in the second reperturbation populations (2R2, 5R2) rapidly decreased to the same apparent equilibrium, i. e. selection. The results for the initial IT6/IH13 populations (cages 1–10, Figures 1 and 2), with *p*(*a*) first increasing and then decreasing could be explained by at least one of the models of two loci epistasis investigated [30], and possibly by other models of interaction among two or more loci. It is unfortunate that these cages were terminated at day 1910, but the predicted trends, with *p*(*a*) in cages 1–5 at apparent equilibrium and *p*(*a*) decreasing in cages 6–10, are expected for some models of two-locus epistasis - see Figure 23 and subsequent discussion in [1].

Over both sets of cages, the results indicate that allele frequencies at the *Est-2* locus are subject to selection, but selection driven by some number of loci, at least some of which act epistatically. We emphasise that these results are specific to the alleles and lines used. Both the *b* (IT6) and *c* (IH13) alleles are in the same limited genetic background (the isochromosome 2 of line IT6, except for the eight unit map region around *Est-2*). Clearly the apparent selection affecting *Est-2* allele frequencies in these cage experiments is not due only to direct selection at this locus. In natural populations with many alleles and greater background genetic variability, more loci and interacting effects are to be expected.

The pair-wise viability estimates (Table 4) show a wide range of outcomes, where for any one allele its genotypes with other alleles may show positive, negative or no selection, and it may be dominant or recessive, or exhibit overdominance. As all lines have the same (or extremely similar) genetic background (except for the small segment around *Est-*2), these relative viabilities are assumed to be due to the specific set of linked genes in each pair of isogenic lines that are included in this small segment (i. e. the genes from one second chromosome of each of the original wild males). The diversity of results for the pair-wise crosses, both for modes of inheritance and relative viability, may suggest selective differences among the *Est-2* alleles themselves. However, relative viability relationships among three or more lines are often inconsistent – for example, IT42 is recessive and favoured over IT16 and IT16 is dominant and favoured over IT8, but there is overdominance for IT42 and IT8. That is, different sets of genes in different lines are determining the apparent modes of inheritance and relative viabilities. This together with the multiplicity of outcomes for these pair-wise viability tests strengthens the conclusion from the perturbation-reperturbation experiments – some number of potentially interacting and closely linked genes are affecting allele frequencies at the *Est-2* locus.

None of these results, however, identifies whether selection is also acting directly at the *Est-2* locus. The EST-2 protein in *D. buzzatii* is encoded by the αE5 ortholog of *D. melanogaster* [31], which is one of 11 genes in the α-esterase cluster encoding functional esterases [32]. This cluster has been mapped to the second chromosome of *D. buzzatii* between cytological bands F5e and F6a, close to but outside the proximal breakpoint of inversion 2j [27]. The natural substrate and function of EST-2 are unknown, but the protein is expressed primarily in the alimentary tract, with high specific activity in the midgut and Malphigian tubules, suggesting a role in digestion or detoxification [23]. *Opuntia* species differ in chemistry of the fresh cladodes [33] and consequently the species may differ in the chemical environment of the rots where larvae develop and feed, and where adults feed. In addition, rot microflora composition varies between rots and over time within rots [34], potentially leading to further variation in the diet chemistry of individual larvae and adults. These chemical differences among *Opuntia* species, among and within rots and among individual diets, and differences among EST-2 variants in detoxification efficiency or targets could be the basis for selection at this locus in natural populations. Supporting this contention, *Est-2* expected heterozygosity and starch gel allele frequencies show significant associations with cactus species in Australian populations [18].

The diversity of patterns of allele frequency change in these experimental populations suggests selection is operating on an interacting set of loci that may include *Est-2*. In any one natural population, general stability of the *Est-2* polymorphism and differences among populations in apparent equilibrium frequencies may result from diverse fitness effects of multiple *Est-2* alleles, and complex interactions of these alleles and alleles of genes at closely linked loci. Analysis of DNA sequence variation across the α-esterase cluster will be necessary to resolve whether there are direct selective differences among the *Est-2* alleles and the type and magnitude of any interactions (epistasis) involving linked loci [5]. Given the homology of the α-esterase clusters in the distantly related species, *D. buzzatii* and *D. melanogaster*, such analysis of both species could inform on the evolutionary history of the region of the α-esterase cluster, and the nature of epistatic interactions among genes in the region.

## Supporting Information

Document S1 Contains Figure S1, S2, and S3.
(DOCX)
