# Supplementary material for: Selection at the Esterase-2 Locus of Drosophila buzzatii? Perturbation-Reperturbation Experiments
Source: PLoS One. 2014 Sep 24;9(9):e108147. doi: 10.1371/journal.pone.0108147 (PMC4176962; doi:10.1371/journal.pone.0108147)

**Document S1. Additional details of Material and Methods**

*(a). Backcrossing IT15 and IH13 into IT6*

In the first generation, virgin females were mated to IT6 males, and virgin female progeny collected. Twelve of these heterozygous females were mated to IT6 males, and virgin female progeny again collected (expected to be one half heterozygous and one half homozygous for the IT6 second chromosome). Twenty of these females were mated individually to IT6 males, and three days later electrophoresed to detect those that were heterozygous. Virgin female progeny were collected from those matings, and single pair backcrossing repeated, again with electrophoresis of the females after egg laying to ensure female progeny were kept only from heterozygous female parents. After 25 backcross generations, male and virgin female progeny were collected, set up as large numbers (about 250) of single pairs, and three days later electrophoresed to detect those matings where both parents were heterozygous. From their progeny, this procedure was repeated to detect pairs homozygous for the IT15 (or IH13) *Est-2* allele. If less than five such matings were obtained, pairs with one parent heterozygous and the other homozygous for the IT15 or IH13 allele were retained, and their progeny again set up as single pairs. For IT15, six independent homozygous pairs were isolated, and eight for IH13. For each of IT15 and IH13, equal numbers of progeny were taken from these matings (or their descendants in the next generation), mixed and set up in four bottles, 24 pairs per bottle, with subsequent generations maintained in this same way. During the backcrossing phase, IT6 was maintained in four bottles, 24 pairs per bottle.

*(b). Initiation of perturbation cages*

Procedures for both sets of cages (IT6/IH13 and IT6/IT15) were the same, but using IT6/IH13 for example, each line was set up in three bottles, 20 pairs of parents per bottle. Virgin progeny were collected and matings set up as: four bottles IT6, four bottles IH13, two bottles IT6 males x IH13 females and two bottles of the reciprocal mating. Virgin progeny were used to initiate two population cages, each with 200 pairs of flies (6-7 days old): (i) initial frequency of IT6 allele = 0.8 – 128 pairs IT6, 32 pairs of heterozygotes from each of the reciprocal crosses, and eight pairs IH13, and (ii) initial frequency of IT6 allele = 0.2 – eight pairs IT6, 32 pairs of heterozygotes from each of the reciprocal crosses, and 128 pairs IH13. The IT6/IH15 cages were founded about four months after the IT6/IH13 cages, but the day of initiation of the egg laying cages in each set was taken as day zero.

**Figure S1**

**IT6/IH13, cages 1 – 5 vs cages 6 - 10**

Test for significance in difference of time course for Cage 1-5 vs 6-10

Chisq = 97.97274 DF = 2 p = 5.314827e-22

Mean *p*(*a*) at 1910 days

CGroup Days predicted.value standard.error est.status

1 1-5 1910 0.6120580 0.03853902 Estimable

2 6-10 1910 0.4921923 0.03853837 Estimable

Comparing mean p(a):

z = 2.23 P = 0.026

So *p*(*a*) in cages 1-5 is significantly higher than those in cages 6-10 at that time point.

Note in the plot below, the time of comparison (1910 days) is shown by the blue marker.


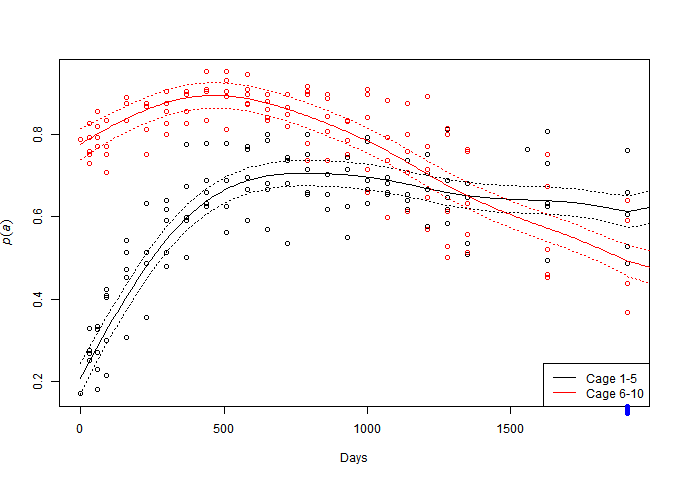


**Figure S2**

**IT6/IH13, cages 1 – 5 vs Cage 2R1**

Test for significance in difference of time course for Cage 1-5 vs 2R1

Chisq = 8.129666 DF = 2 p = 0.01716586

Mean *p*(*a*) at 1910 days

CGroup Days predicted.value standard.error est.status

1 1-5 1910 0.6075442 0.03762711 Estimable

2 2R1 1910 0.5950986 0.06127945 Estimable

Comparing mean p(a):

z = 0.17 P = 0.85

So Cage 2R1 is not significantly different from Cages 1-5.

Note in the plot below, the time of comparison (1910 days) is shown by the blue marker.


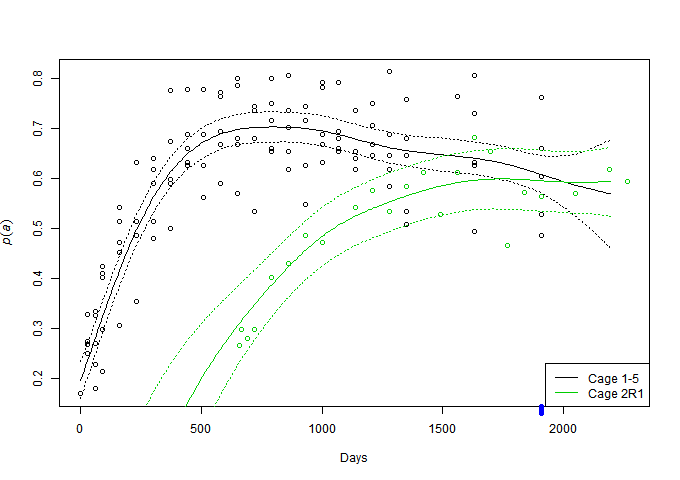


**Figure S3**

**IT6/IT15, cages 11 – 15 (excluding cage 13) vs cages 16 – 20 (excluding cage 19)**

Test for significance in difference of time course for Cage 11-15 vs 16-20

Chisq = 30.75894 DF = 2 P = 2.093061e-07

Mean responses at 1155 days

CGroup Days predicted.value standard.error est.status

1 11-15 1155 0.02574230 0.03079636 Estimable

3 16-20 1155 0.06808322 0.01898194 Estimable

z = 1.19 P = 0.23

Mean responses at 1645 days

CGroup Days predicted.value standard.error est.status

2 11-15 1645 0.08729749 0.15742292 Estimable

4 16-20 1645 0.09131163 0.02280961 Estimable

z = 0.03 P = 0.98

So no significant differences at these two points, nor at any intervening time; however, standard errors of predictions are getting quite large.

Note in the plot below, the times of comparison (1155 days, 1645 days) are shown by the blue markers.


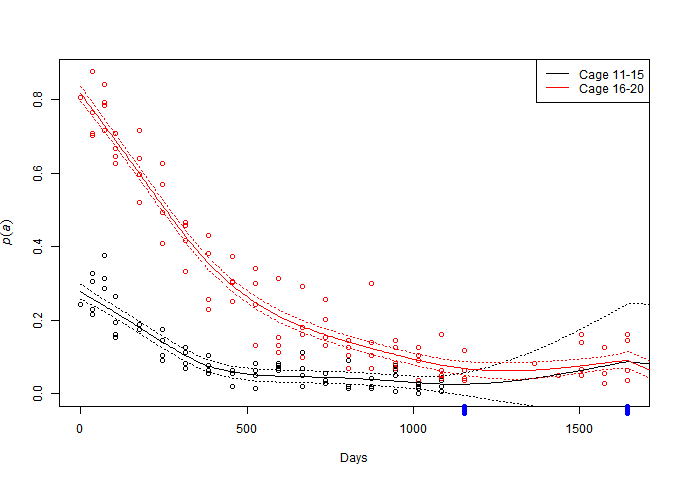

Supplement: Document S1 — Contains Figure S1, S2, and S3. (DOCX) [file pone.0108147.s001.docx]
